# Role of MR Morphology and Diffusion-Weighted Imaging in the Evaluation of Meningiomas: Radio-Pathologic Correlation

**DOI:** 10.31729/jnma.3968

**Published:** 2019-02-28

**Authors:** Kajan Ranabhat, Suresh Bishokarma, Prity Agrawal, Pratyush Shrestha, Ram Kumar Ghimire, Rajesh Panth

**Affiliations:** 1 Department of Radiology, Upendra Devkota Memorial National Institute of Neurological and Allied Sciences, Bansbari, Kathmandu, Nepal; 2 Department of Neurosurgery, National Institute of Neurological and Allied Sciences, Bansbari, Kathmandu, Nepal; 3 Department of Neuropathology, National Institute of Neurological and Allied Sciences, Bansbari, Kathmandu, Nepal

**Keywords:** *apparent diffusion coefficient*, *atypical*, *histopathology*, *meningioma*, *MR morphology*

## Abstract

**Introduction:**

Preoperative differentiation of benign, atypical and malignant meningiomas would significantly help in surgical planning and treatment. The aim of this study is to look at radio-morphologic behavior of various histopathological types and grades of meningiomas and their diffusion characteristics.

**Methods:**

We performed an analytical cross-sectional study including all patients operated on for meningiomas at our hospital during January 2016 to July 2018. We studied 38 meningiomas in 38 patients aged 14 to 73 years old. All patients underwent MRI prior to surgery, including diffusion-weighted sequences, in a 1.5T scanner. Signal intensity in T2-weighted images, diffusion-weighted images (b=0, 90 and 1,000), and Apparent Diffusion Coefficient maps within the tumors and in the normal parietal white matter as a reference were evaluated. In the histological study, cellularity, proliferation index, histological grade, and cerebral invasion were evaluated.

**Results:**

There was female predilection with male:female ratio of 1:2.4. Most meningiomas were supratentorial with most common origin being parafalcine and convexity. Of the 38 meningiomas, 31 were WHO grade I, 6 were WHO grade II (atypical) and one was WHO grade III (anaplastic). Among various tumors' behaviors, incomplete CSF cleft, pial invasion and parenchymal invasion were significantly high in high-grade tumors. Similarly, tumors showing pial invasion, breached tumor-brain interface, no capsular enhancement and parenchyma invasion showed significantly low NADC. Mean ADC value was 0.722±7.7x10^−3^ mm^2^/s (normalized ADC 0.9±0.1) in the atypical group and 0.876±24.56x10^−3^ mm^2^/s (normalized ADC 1.11±0.31) in the typical group. No statistically significant differences of ADC/NADC were found between histologic subtypes. Two subtypes of typical meningiomas, metaplastic and angiomatous meningioma had the highest values in the ADC maps.

**Conclusions:**

MR morphology like pial invasion, breached tumors brain interface, parenchymal invasion can predict aggressiveness and atypical nature of meningiomas. Meningioma shows moderately restricted diffusion. The signal on the ADC map is associated with tumors cellularity and aggressiveness suggesting its usefulness for predicting the histological grade.

## INTRODUCTION

Meningiomas comprise about 14% to 20% of all intracranial tumors.^1^ Most meningiomas are benign and even asymptomatic. Atypical meningiomas make 7.2% and rarer malignant ones constitute 2.4% of all meningiomas.^2^ These have higher propensity of recurrence with aggressive growth pattern thereby increasing patient morbidity and mortality.^2^

MRI is modern day imaging modality of choice for meningioma. Preoperative differentiation of benign and atypical meningiomas significantly helps in surgical and treatment planning. This however is not reliably accomplished assessing the imaging features on routine MRI alone.^3^ Diffusion-weighted imaging (DWI) has been used in primary brain neoplasms. Correlations between apparent diffusion coefficient (ADC) values, tumors cellularity, and tumors grade have been made. Use of DWI to monitor treatment response has been evaluated.^4,6^ However role of DWI in the diagnosis or prognosis of extra-axial neoplasms is unclear.

This study aims to examine various morphometric and signal characteristics of meningiomas on conventional sequences and correlate diffusion coefficient with histopathology. We hypothesized certain conventional MRI characters and diffusion constant may help distinguish benign and atypical meningiomas.

## METHODS

Neuroradiology imaging and neuropathology database of all consecutive cases of meningioma that were admitted and underwent surgical resection at Upendra Devkota Memorial National Institute of Neurological and Allied Sciences from January 2016 to July 2018 were retrospectively reviewed. Atypical and malignant meningiomas were diagnosed based on the WHO classification of grades II and III tumors, respectively. The exclusion criteria were previous radiotherapy or radiosurgery, preoperative trans-arterial embolization, and incomplete or uninterpretable preoperative MRI studies. Institutional review board of the respective institute approved this study. A total of 38 patients were enrolled, including 31 (75%) with Grade I meningiomas and 7 (25%) with high-grade (Grade II or III) meningiomas.

Preoperative MRI was available for each patient and was performed using a 1.5-T MR unit (Magnetom, Essenza, Siemens). The MRI protocol was TR 3900 msec, TE 111 msec, matrix size 230x230, section thickness 5 mm, and intersection gap 0.21 mm with b-values of 0, 90 and 1000 s/mm^2^ in 3 orthogonal directions. Routine images of the whole brain, including spin echo T1-weighted images, spin echo T2-weighted images, and fluid-attenuated inversion recovery (FLAIR) images were obtained. Spin echo contrast-enhanced T1-weighted images were obtained in the coronal, sagittal, and axial planes after intravenous Gadolinium administration (0.1 mmol/kg body weight). Diffusion-weighted imaging (DWI) was acquired in the axial plane using a singleshot, spin echo, echo planar imaging sequence.

Signal intensities of the meningiomas on T1- and T2-weighted imaging were recorded as hypointense, isointense, or hyperintense relative to the intensity of the gray matter. Meningiomas with distinct peritumoral rims and CSF clefts, which were hypointense on T1-weighted imaging and hyperintense on T2-weighted imaging, were defined as clear tumors-brain interface. In contrast, tumors without distinct borders were defined as unclear tumors-brain interface.

The pattern of contrast enhancement after Gd administration was divided into homogeneous or heterogeneous. Intratumoral cystic change, defined as an area of hyperintensity on T2-weighted imaging and hypointensity on T1-weighted imaging without contrast enhancement, was regarded as heterogeneous enhancement in this study. Capsular enhancement was defined as the entire enhanced layer at the tumors-brain interface and was categorized as positive or negative. The presence of brain edema was judged as a hyperintense extension adjacent to tumors on T2-weighted imaging and was judged as positive or negative.

The DWI was visually inspected and classified as hyperintense, isointense, or hypointense in comparison with normal white matter. According to the particular site of origin, the location of each intracranial meningioma was divided into convexity, tentorial and bony on which dura of origin is predominantly based. The image interpretation of each MRI feature was described and confirmed by 2 experienced radiologists.

All data were analyzed using the SPSS statistical program version 20. The association between radiological features of MRI along with patient age and sex and the histopathological grade of meningiomas were examined by univariate and multivariate analyses. Logistic regression was used to identify significant factors that were predictive of high-grade meningiomas.

## RESULTS

Mean age of the patients was 43.5 years with age range of 14-73 years. Out of 38 total meningiomas, 11 (28.9%) were found in male and 27 (71.1%) were found in female.

Laterality of the meningiomas was 17 (44%) on the right and 16 (42%) on left with 2 (5.3%) extending to both sides and 3 (7.9%) in midline. Supratentorial location was observed in 30 (78.9%) patients while infratentorial among 8 (21.1%) patients. Most common meningioma was convexity meningiomas 11 (28.9%) followed by parafalcine meningiomas 8 (21.1%) and skull base 8 (21.1%) (Table 1).

**Table 1.** Location of meningiomas.

Location	n (%)
Convexity	11 (28.9)
Parafalcine	8 (21.1)
Skull Base	8 (21.1)
Tentorial	5 (13.2)
Sphenoid wing	4 (10.5)
Olfactory Groove	1 (2.6)
Infratentorial	1 (2.6)
Total	38 (100)

In the supratentorial group, most common location was frontal 13 (34.2%) followed by temporal 5 (13.2%). Mean tumors burden was 39.15±36.74 cc at the time of presentation with volume ranging from 3.29 cc to 134 cc. Twenty-four (63.2%) of the tumors were isointense, 11 (28.9%) were hypointense and 3 (7.9%) were heterointense in T1-weighted sequences. Thirteen (34.2%) of the tumors displayed isointense, 11 (28.9%) hyperintense, 8 (21.1%) showed heterointense and rest of the tumors showed hypointese signal in T2-weighted images. Likewise, 13 (34.2%) of tumors showed isointense and hyperintense signal each in FLAIR images.

Thirty-three (86.8%) of tumors did not show any hemorrhage in SWI whereas 5 (13.2%) of the tumors showed hemorrhage of various degree. Twenty-three (60.5%) of tumors showed sharp tumors margins and rest had fuzzy tumors margins. Lobulations were present in half of the tumors. Nodularity was present in 25 (65.8%) of the tumors. Thirty-five (92.1%) of the tumors showed CSF cleft. Mild parenchymal edema was present in 26 (68.4%) of the tumors and 2 (5.3%) tumors had moderate edema. Nine (23.7%) tumors involved cerebral venous sinuses, out of which 2 (5.3%) were abutting the sinus, 6 (15.8%) were compressing the sinuses and 1 (2.6%) had invaded the sinus.

Homogenous contrast uptake was showed by 27 (71%) of tumors and 10 (26.3%) showed heterogenous enhancement. Thirty (78.9%) of meningiomas showed complete capsular enhancement whereas 8 (21.1%) showed either incomplete breached capsular enhancement or no capsular enhancement. Flow voids were present in 13 (34.2%) of the tumors and cysts were present in 2 (5.3%) of the tumors. Likewise, tumors necrosis was present in 5 (13.1%) of the tumors. Enhancing dural tail was present in 100% of the tumors with hyperostosis of overlying calvarian in 10 (26.3%) of the tumors and erosion was present in 9 (23.7%) of the tumors. Rest of the tumors caused either no change or were not adjacent to bone.

Pial invasion was present in 17 (44.7%) of the tumors. Tumors brain interface was intact in 26 (68.4%) of the tumors and breached in the rest 12 (31.6%). Similarly, parenchymal invasion was also seen in 12 (31.6%) of the tumors.

**Figure 1.**
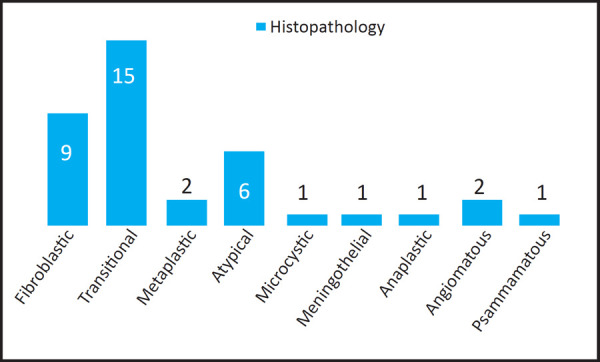
Histologic sub-types of meningiomas.

Histopathology revealed various sub-types of meningiomas (Figure 1). Low grade tumors were seen among 31 (81.6%) cases while 7 (18.4%) of tumors were high grade. Among high grade, 6 meningiomas were atypical and 1 was anaplastic meningioma.

There was no significant difference in nodularity, lobulations and tumors margin between low grade and high-grade tumors. Likewise, no significant difference in parenchymal edema was seen between these two groups. Capsular enhancement, flow voids, venous sinus involvement and tumors brain interface also showed no significant difference between low grade and high-grade tumors. However, there was significant difference in pial invasion and parenchymal invasion between these two groups with pial invasion and parenchymal invasion significantly higher in high-grade meningiomas. Incomplete CSF cleft also showed to be significantly high in high-grade meningioma (Table 2).

**Table 2.** Correlation of tumors grades with various tumors characters.

Variables	Characteristics	Total n (%)	Low (Grade I) n (%)	High (Grade II & III) n (%)	P
Tumors margin	Sharp	29 (76.3)	24 (77.4)	5 (71.4)	0.736
	Fuzzy	9 (23.7)	7 (22.6)	2 (28.6)	
Lobulation	Absent	19 (50)	15 (48.4)	4 (75.1)	0.676
	Present	19 (50)	16 (51.6)	3 (42.9)	
Nodularity	Absent	25 (65.8)	19 (61.3)	6 (85.7)	0.219
CSF Cleft	Present	13 (34.2)	12 (38.7)	1 (14.3)	0.046
	Absent	1 (2.6)	0	1 (28.6)	
	Complete	35 (92.1)	30 (96.8)	5 (71.4)	
	Incomplete	2 (5.3)	1 (3.2)	1 (14.3)	
Pial Invasion	None	21 (55.3)	20 (64.5)	1 (14.3)	0.012
	Invasion	17 (44.7)	11 (35.5)	6 (85.7)	
Parenchymal invasion	None	26 (68.4)	24 (77.4)	2 (28.6)	0.022
	Invasion	12 (31.6)	7 (22.6)	5 (71.4)	
Necrosis	Absent	33 (86.8)	28 (90.3)	5 (71.4)	0.182
	Present	5 (13.1)	3 (9.7)	2 (28.6)	
Edema	No edema	10 (26.3)	8 (25.8)	2 (2.8)	0.472
	Mild	26 (68.4)	22 (71)	4 (57.1)	
	Marked	2 (5.3)	1 (3.2)	1 (14.3)	
TB Interface	Intact	26 (68.4)	22 (71)	4 (57.1)	0.656
	Breached	12 (31.6)	9 (29)	3 (42.9)	
Capsular enhancement	None	7 (18.4)	4 (12.9)	3 (42.9)	0.065
	Enhanced	31 (81.6)	27 (87.1)	4 (57.1)	
Flow Void	None	25 (65.8)	19 (61.3)	6 (85.7)	0.20
	Few	10 (26.3)	10 (32.3)	0	
	Abundant	3 (7.9)	2 (6.5)	1 (14.3)	
Sinus	Uninvolved	29 (76.3)	23 (74.2)	6 (85.7)	0.852
	Abutting	2 (5.3)	2 (6.5)	0	
	Compressed	6 (15.8)	5 (16.1)	1 (14.3)	
	Invaded	1 (2.6)	1 (3.2)	0	

Tumors with lower NADC values showed significantly more pial invasion, parenchymal invasion, breached tumors brain interface and breached capsular enhancement. Significant difference in NADC value was seen in various histologic subtypes of meningioma. However, low ADC value was seen in atypical meningioma (0.618x10^−3^ mm^2^/s), anaplastic meningioma (0.645x10^−3^ mm^2^/s) and meningothelial meningioma (0.727x10^−3^ mm^2^/s) and highest ADC was seen in metaplastic meningioma.

Mean ADC in low-grade tumors was 0.722x 10^−3^ mm^2^/s and low grade was 0.876.6x10^−3^ mm^2^/s (Table 3). No significant difference in tumors grade was seen between meningiomas arising in skull base and those arising at sites other than skull base.

**Table 3.** Correlation of Normalized ADC with variables.

Variables	Characteristics	Total n (%)	NADC >1 n (%)	NADC<1 n (%)	P
Tumors margin	Sharp	29 (76.3)	20 (90.9%)	9 (56.3)	0.013
	Fuzzy	9 (23.7)	2 (9.1%)	7 (43.8)	
Lobulation	Absent	19 (50)	14 (63.6%)	5 (31.3)	0.049
	Present	19 (50)	8 (36.4%)	11 (68.8)	
Nodularity	Absent	25 (65.8)	15 (68.2)	10 (62.5)	0.715
	Present	13 (34.2)	7 (31.8)	6 (37.5)	
CSF Cleft	Absent	1 (2.6)	0	1 (6.3)	0.107
	Complete	35 (92.1)	22 (100)	13 (81.3)	
	Incomplete	2 (5.3)	0	2 (12.5)	
Pial Invasion	None	21 (55.3)	17 (77.3)	4 (25)	0.001
	Present	17 (44.7)	5 (22.7)	12 (75)	
Parenchymal invasion	None	26 (68.4)	19 (86.4)	7 (43.8)	0.005
	Invasion	12 (31.6)	3 (13.6)	9 (56.3)	
Necrosis	Absent	33 (86.8)	21 (95.4)	12 (75)	0.066
	Present	5 (13.2)	1 (4.6)	4 (25)	
Edema	No edema	10 (26.3)	7 (31.8)	3 (18.8)	0.187
	Mild	26 (68.4)	15 (68.2)	11 (68.8)	
	Marked	2 (5.3)	0	2 (12.5)	
TB Interface	Intact	26 (68.4)	19 (86.4)	7 (43.8)	0.005
	Breached	12 (31.6)	3 (13.6)	9 (56.3)	
Capsular enhancement	None	7 (18.4)	2 (9.1)	5 (31.3)	0.082
	Enhanced	31 (81.6)	20 (90.9)	11 (68.8)	
Flow Void	None	25 (65.8)	16 (72.1)	9 (56.3)	0.501
	Few	10 (26.3)	5 (22.7)	5 (31.3)	
	Abundant	3 (7.9)	1 (4.5)	2 (12.5)	
Sinus	Uninvolved	29 (76.3)	16 (72.7)	13 (81.3)	0.788
	Abutting	2 (5.3)	1 (4.5)	1 (6.3)	
	Compressed	6 (15.8)	4 (18.2)	2 (12.5)	
	Invaded	1 (2.6)	1 (4.5)	0	
Meningioma Type	Fibroblastic	9 (23.7)	6 (27.3)	3 (18.8)	0.249
	Transitional	15 (39.5)	10 (45.5)	5 (31.3)	
	Metaplastic	2 (5.3)	1 (4.5)	1 (6.3)	
	Atypical	6 (15.8)	2 (9.1)	4 (25)	
	Microcytic	1 (2.6)	0	1 (6.3)	
	Meningothelial	1 (2.6)	0	1 (6.3)	
	Anaplastic	1 (2.6)	0	1 (6.3)	
	Angiomatous	2 (5.3)	2 (9.1)	0	
	Psammamatous	1 (2.6)	1 (4.5)	0	
Grade of Meningioma	Low	31 (81.6)	20 (90.9)	11 (68.8)	0.082
	High	7 (18.4)	2 (9.1)	5 (31.3)	

## DISCUSSION

Predicting pre-surgical histopathological grade of meningioma is helpful in appropriate treatment plan. The association between specific radiological features and aggressive biological behavior has been studied separately by other investigators.^7–10^ Our study attempts to incorporate the use of diffusion restriction and other radio-morphological findings in predicting grades of meningioma. As established by previous studies, in this study as well, there is female predilection of meningioma with male to female ratio of 1:2.4. The finding that age was a risk factor for high-grade meningioma is controversial. It has been reported that age is an independent variable in predicting tumors recurrence and degree of differentiation according to previous reports.^11^ However, this is not supported by our study result. Heterogeneous MRI enhancement after Gd injection is associated with uneven distribution of tumor cells or even ischemic necrosis, hemorrhage, cystic degeneration, accumulation of tumor cell secretion, and evidence of rapid tumor expansion, the biological features of malignant tumors.^2,12^ Several reports have stated that Grade II and III meningiomas have significantly more intratumoral cystic changes compared with Grade I meningiomas.^3,13^ In the present study, heterogeneous enhancement, as well as the presence of an intratumoral cyst, was an important factor predictive of high-grade meningioma, consistent with previous studies.

The interface between the tumor and the brain is determined by the expression of a peritumoral rim. A clear peritumoral rim indicates the presence of a physiological barrier between the meningioma and brain parenchyma and an unclear peri-tumoral rim suggests tumor adhesion and invasion of the surrounding brain tissue, the logical feature of aggressive biological behavior.^14,15^ As in previous reports, an unclear tumor-brain interface was a significant indicative factor in predicting high-grade meningiomas in both univariate and multivariate analyses in our study.

Likewise, a positive capsular enhancement, defined as the enhanced layer at the tumor-brain interface, was another identified predictor in our study. Meningiomas with unclear tumors-brain interface had negative capsular enhancement or partial loss of capsular enhancement. This means that unclear tumor-brain interface is a negative confounder in determining the association between positive capsular enhancement and high-grade meningioma. This fact is supported by the findings of this study that absence or incomplete capsular enhancement, unclear tumor-brain interface, pial invasion and parenchymal invasion were significantly higher in high-grade meningioma. In addition to this, loss of CSF cleft also showed significant difference being higher in high-grade meningioma.

Alteration in physiological barrier created by the arachnoid membrane and CSF cleft between the tumor and the adjacent brain parenchyma can be the reason for parenchymal edema related to meningioma.^16,18^ Nakano et al. reported that the invasive pattern of tumor-brain interface including irregular tumors margins, loss of the peri-tumoral rim, and hyperintensity of the tumor on T2-weighted imaging was associated with meningioma-related brain edema. However, several studies showed no significant correlation between histological subtypes of meningiomas and peri-tumoral brain edema.^2,19^ In the present study as well, no statistically significant difference in brain edema was found between benign and high-grade meningiomas.

Some studies have reported that meningiomas with skull base locations were associated with a decreased risk of being high grade.^7,20^ We observed in our study that there was no significant difference in the grade between non-skull base meningiomas and skull base meningiomas.

We found that the mean ADC value and NADC ratio were lower (i.e. relatively restricted diffusion) in atypical/ malignant meningioma than in benign tumors (Figure 4). Two previous studies showed similar results.^1,8^ There are various explanations of this difference in literature like increased tumor cellularity, tumor matrices, fibrous or gliotic tissues, or a combination of these factors. ^12^ Water diffusion in biologic tissue is highly dependent on the ratio of intracellular to extracellular space, and increased cellularity with higher mitotic activity and high nucleus-to-cytoplasm ratio in high-grade tumors may decrease the fraction of extracellular space, thus restricting net water diffusion.^21–23^ Many previous studies have tried to establish the use of absolute apparent diffusion coefficient (ADC) in differentiating the histopathological grade of meningiomas.^24–30^ The absolute cut-off and reliability of ADC measurement is controversial, with different b values, areas of measurement (tumors peduncle, peripheral part of the tumor, and central region of the tumor), and methods of measurement (minimum ADC, mean ADC, maximum ADC, and normalized ADC) used in respective studies.

The measurement of absolute ADC values may vary across different scanners, and the NADC ratio may be a consistent parameter to use. The NADC ratio minimizes the differences in absolute ADC values that may be obtained with different DW imaging sequences and hardware configurations, thereby eliminating inter-image variability. This study also identified that meningiomas with lower ADC/NADC had significant association with breach in tumor brain interface, capsular enhancement, pial invasion, parenchymal invasion as well as heterogenous pattern of contrast enhancement.

In clinical practice, predicting meningiomas with a lower probability of advanced histopathological grade; selective resection balanced against the risk of a surgical procedure is recommended. Otherwise, more aggressive resection, and even dura substitution, should be considered for those with a higher probability of a high-grade meningioma.

Our study has some limitations. First, this is a retrospective study, and further prospective reports are needed to test the validity of our prediction model. Second, the patient population comes from a tertiary medical care center, and therefore the sample might not be representative of the entire population. Third, the description of imaging findings is somewhat subjective, with the possible existence of intra-observer and interobserver variability. Fourth and most importantly, the sampling bias of 7 patients with high-grade meningioma and 31 patients with benign meningioma would have influenced the probability calculation.

## CONCLUSIONS

This study shows some important association between few MR morphologic characters with aggressiveness and grade of meningioma. High grade/atypical meningiomas had loss of capsular enhancement, breach in tumor brain interface, loss of CSF cleft and pial/parenchymal invasion. DWI findings also correlated with above mentioned MR parameters. Atypical/Grade II/III tumors had statistically significant lower ADC_mean_ values than grade I meningiomas.

ADC_mean_ can be helpful in preoperative distinction between benign and atypical/malignant meningioma.
